# Relationships Between Cross-Cultural Adaption, Perceived Stress and Psychological Health Among International Undergraduate Students From a Medical University During the COVID-19 Pandemic: A Moderated Mediation Model

**DOI:** 10.3389/fpsyt.2021.783210

**Published:** 2021-12-10

**Authors:** Xiaobin Wang, Lu Lu, Xuehang Wang, Min Qu, Lulu Yuan, Yuqin Gao, Bochen Pan

**Affiliations:** ^1^Center for Reproductive Medicine, Department of Obstetrics and Gynecology, Shengjing Hospital of China Medical University, Shenyang, China; ^2^China Medical University-The Queen's University of Belfast Joint College, China Medical University, Shenyang, China; ^3^International Education School, China Medical University, Shenyang, China; ^4^Liaoning Provincial Key Laboratory of Oral Diseases, School and Hospital of Stomatology, China Medical University, Shenyang, China

**Keywords:** cross-cultural adaption, perceived stress, optimism, resilience, psychological health, international undergraduates, COVID-19

## Abstract

International university students may be at greater risk for developing psychological problems due to the unique stressors in them, particularly during the COVID-19 pandemic. The purpose of present study is to propose and test a moderated mediation model that would illuminate the underlying relationships of cross-cultural adaption, perceived stress and psychological health as well as the moderating effect of optimism and resilience among international medical undergraduates in China during the COVID-19 pandemic. The study was conducted via a web-based survey in November 2020. Electronic informed consents were obtained from all participants. A total of 453 students including 233 males and 220 females aged 18 to 28 years with an average age of 22.09 (*SD* = 2.73) completed the questionnaires. Symptom Checklist 90, the measurement of cross-cultural adaption, the Perceived Stress Scale, the Life Orientation Test-Revised and the Resilience Scale were used for the survey. Results for the moderated mediation model testing revealed that cross-cultural adaption significantly and negatively associated with the Global Severity Index (GSI) of the Symptom Checklist 90 (β = −0.24, *P* < 0.01), and perceived stress partially mediated the relationship. Optimism (β = −0.29, *P* < 0.01) and confidence in COVID-19 control (β = −0.19, *P* < 0.01) had direct negative effects on perceived stress. Furthermore, optimism and resilience negatively moderated the indirect effect of cross-cultural adaption on psychological health through perceived stress. Findings of this study suggest that university educators ought to promote or make use of programs that cope with stress and boost optimism and resilience in order to support students not only adapt well to a new culture, but also keep good psychological health during the period of COVID-19 pandemic.

## Introduction

Along with the trend of globalization, more and more students choose to receive their higher education abroad. Proportion of international student within the campus has been recognized as one of the important indicators in various world university rankings, and the international students have already been an essential part of the higher education worldwide. With the rapid development of economy and the expanded international influence, China has attracted more and more international students since the twenty first century. In 2015, nearly 400,000 international students from 202 countries and regions were studying in China, an increase of 50% over in 2010 (1). Among them, the number of students specialized in medicine, engineering and science increased most remarkably (1). In 2018, this figure increased to 492,200, and China has become the largest destination for overseas study in Asia (1). However, for the international students, studying and growing up in another culture can be very challenging, and at the time of public crisis such as the COVID-19 pandemic, the challenge they are facing could be overwhelming.

During the COVID-19 pandemic, control and prevention measures such as lockdown, reduction of social contact and changes of teaching mode interrupted the students' daily life, hampered their studies and negatively impacted their psychological health (2). Moreover, previous studies revealed that the international students were more prone to psychological problems because, compared with their domestic peers, they lacked resources to counteract stress and had to cope with more challenges unique to them, such as the language barrier and culture shock (3, 4). However, most of the previous studies were done in western countries, and the research in Asia has been limited, especially during the COVID-19 pandemic. According to the results of a recent survey to the international students, 73.4, 76.6, and 58.5% of the participants had depression, anxiety and stress symptoms, respectively, during the pandemic, and male students presented more symptoms of depression and anxiety than females due to their more risk-taking behaviors (2). As international medical students will be incorporated into the future work force safeguarding the health of mankind, their well-being especially their mental health during the pandemic should arouse our attention. Therefore, the purpose of this study is to investigate the psychological health and the related factors of this student population in order to provide proper help to them during the period of COVID-19 pandemic.

Cross-cultural adaption is one of the challenges that international students have to cope with. Many researchers have examined the cross-cultural adaption or other related concepts in the realm of acculturation and cultural shock. The term cross-cultural adaption has been used to indicate a wide range of definitions which include a host language ability, a feeling of acceptance, the nature and extent of interaction with host nationals, or the acquisition of culturally acceptable behaviors (5). Cross-cultural adaption is often described as a stressful process for international students (6). Previous studies showed that the international undergraduates had more psychological problems, and failure in cross-cultural adaption might be an important stressor (7–9). According to Berry's acculturative stress theory, high levels of acculturation stress exceeding the individual's coping capacity is considered detrimental and thought to be the main mechanism for psychological distress among the immigrant population (10). Thus, stress perception might be an essential contributor to the relationship between cross-cultural adaption and psychological health.

With the emergence of the scientific field of positive psychology (11), the interest in understanding individuals from a positive psychological perspective has become a current development in higher education practice (12). Although there is still limited research on positive psychological health among undergraduates in universities (13), strong evidence exists supporting the relationship between positive psychology and psychological health of employees in organizational settings (14).

As one of the important positive psychological resources, optimism is defined as positive expectation of future success that creates renewed efforts to attain goals despite of any adversity that may be anticipated or created by stressful experiences (15). Optimism has been linked to better psychological health (16) and physical health (17), and many studies have documented optimism's protective effects against stress, which suggests that optimism may moderate the development of psychological problems in response to stress (18). Furthermore, optimism has also been found to be useful in dealing with psychological stressors (19). A meta-analysis examining experimental studies found that optimism was a significant moderator of the effects of stressors on psychological health, such that individuals with lower optimism typically showed the greatest benefit from the intervention (20). Therefore, optimism may moderate the impact of both stressors and stress on psychological health.

On the other hand, as another important positive psychological resource, resilience has been characterized by the ability to bounce back from negative emotional experiences and by flexible adaption to the changing demands of stressful experience (21). Individuals who have the ability to adapt and bounce back in adverse situations tend to exhibit strength in making realistic plans and taking necessary actions. It is recognized that resilience was an important protective factor against the development of psychiatric disorders in the face of continued adversity (22). Previous studies revealed that resilience might help undergraduates coping effectively with the stress in university life (23). Thus, resilience may be a moderator in the relationship between stress and psychological health.

**Figure 1:**
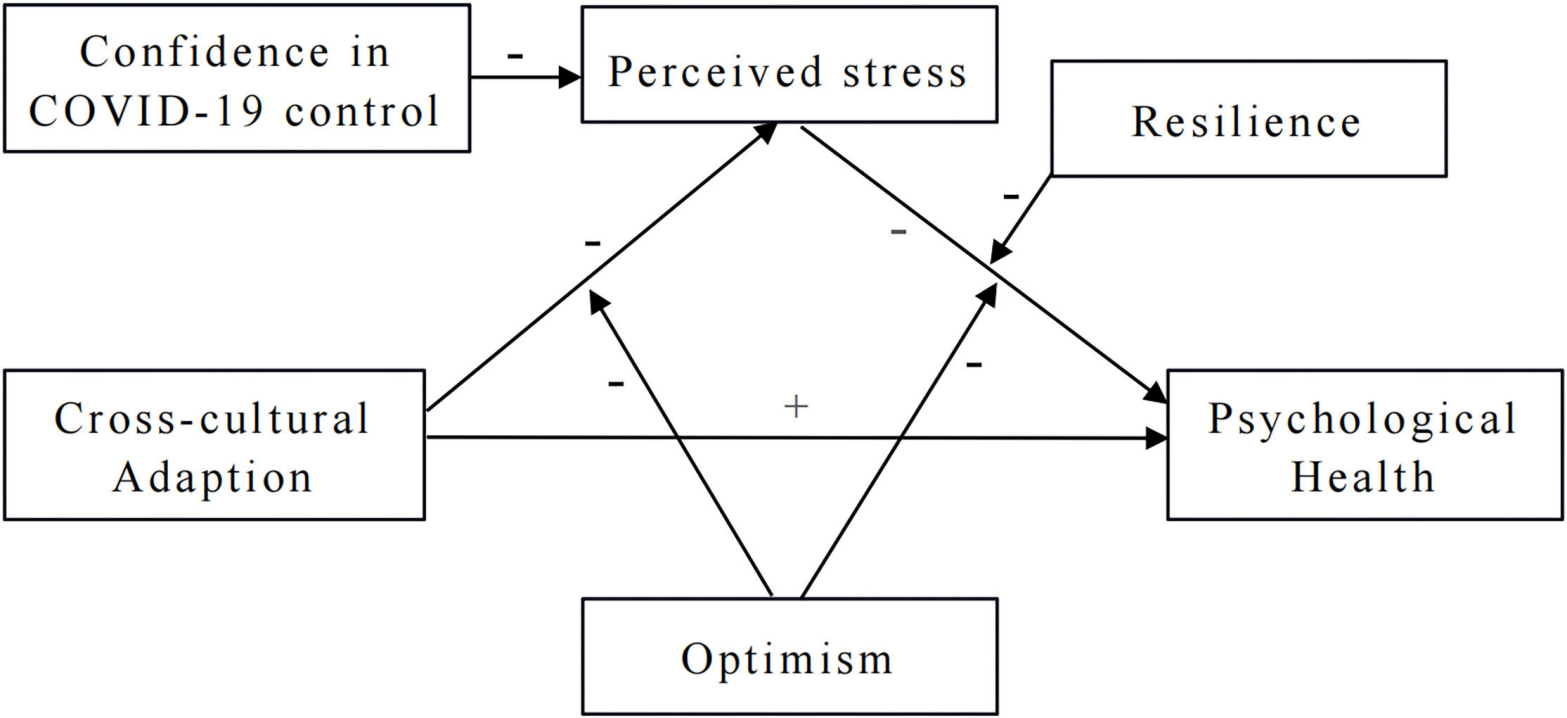
The conceptual diagram of moderated mediation model.

However, little research has been done to study the mechanisms of the relationship between cross-cultural adaption and psychological health including the mediating or moderating variables (24). Therefore, the present study aims to test a moderated mediation model that proposes the underlying relationships of cross-cultural adaption, perceived stress and psychological health among international medical undergraduates from a Chinese university during the COVID-19 pandemic. In addition, the moderating effect of optimism and resilience will also be explored. For that purpose and based on the literature review, we formulate the following hypotheses: (H1) Perceived stress mediated the relationship between cross-cultural adaption and psychological health; (H2) Optimism moderated the relationship between cross-cultural adaption and perceived stress; (H3) Optimism moderated the relationship between perceived stress and psychological health; (H4) Resilience moderated the relationship between perceived stress and psychological health. The proposed moderated mediation model is depicted in the conceptual diagram in Figure 1.

## Materials and Methods

### Study Design and Subjects

The study was conducted via a web-based survey in November 2020. A cluster sampling was used and the international undergraduates enrolled at one Chinese university (China Medical University) were the target population for the study. The inclusion criteria required that each participant was under the period of study and could access to the internet. Ethics approval from the Research Ethics Committee at China Medical University (2020-25) was obtained on March 16th, 2020. There were a total of 945 international undergraduates who met the criteria, and the emails were sent to them for invitation to the survey. The email briefly detailed the purpose of the study and invited the students to participate with a link to the online survey. The first page of the online survey included a brief description of the study and an informed consent letter, in which the participants were informed about their rights to decline participation and measures to protect their confidentiality. If the students agreed to the informed consent, they could then begin the questionnaires. In the end, a total of 453 international medical undergraduates out of 500 responses completed the questionnaires, with a response rate of 47.94%. The sample consisted of 233 (51.43%) males and 220 (48.57%) females. Their ages ranged from 18 to 28 years, with an average age of 22.09 years (standard deviation = 2.73). The students were originally from 49 countries, of which 84.98% were from Asia, 7.95% from Africa, 3.09% from Europe, 2.21% from North America, and 1.77% from Oceania.

### Measurements

#### Measurement of Psychological Health

The Symptom Checklist 90 (SCL-90) has been widely used to assess the psychological functioning not only in psychiatric patients but also in non-clinical population (25). Therefore, it was used to evaluate the psychological health of international undergraduates in this study. The SCL-90 is a multidimensional symptom self-report clinical rating scale, and contains 90 items in which respondents are to rate their degree of distress on each item according to a five-point Likert scale ranging from not at all to extremely. Eighty-three items reflect nine symptoms dimensions, which are Somatization (12 items), Obsessive-Compulsive (10 items), Interpersonal Sensitivity (9 items), Depression (13 items), Anxiety (10 items), Hostility (6 items), Phobic Anxiety (7 items), Paranoid Ideation (6 items), and Psychoticism (10 items) (26). The Global Severity Index (GSI) of the SCL-90 is regarded as the best single indicator to reflect overall symptom severity, because it combines information on both numbers of symptoms and intensity of distress (26). The GSI and the dimension scores can be calculated by summing up all the distress scores or the ones belong to a specific dimension, and then divided by 90 or the number of items to the specific dimension. A number of studies have been conducted demonstrating the reliability and the validity of this instrument (27, 28), and a Cronbach's alpha of 0.98 was found in this study.

#### Measurement of Cross-Cultural Adaption

Cross-cultural adaption of the international undergraduates was measured by 6 questions regarding the language barrier and its negative influence on their life and study, the negative impact of the cultural differences, and whether they were already adapted to the study and life at the university. Response options are presented along a four-point Likert-type scale ranging from not at all to very much, and a higher summative score (negative items are reverse-scored) represents a higher level of cross-cultural adaption. The Cronbach's alpha for this sample was 0.75.

#### Measurement of Confidence in COVID-19 Control

Confidence in COVID-19 control was measured by 5 questions regarding if the participant is taking personal protective measures and the confidence in the personal protective measures, the confidence in the knowledge and the ability to protect oneself from being infected, and if the participant is confident that the outbreak will eventually be contained. Participants answered 1 (not at all) to 4 (very much) to the questions, and a higher total score represented a higher level of confidence in the COVID-19 control. The Cronbach's alpha was 0.72 in this study sample.

#### Measurement of Perceived Stress

The Perceived Stress Scale (PSS) was used to obtain an indication of the extent to which the international undergraduates perceived situations in their life to be stressful. It is the most widely used psychological measurement for the perception of stress, and requires respondents to indicate how frequently in the past month they had perceived their life to be overwhelming (29). The scale consists of 10 items, and a five-point Likert scale ranging from 0 (never) to 4 (very often) is used for answering statements. The PSS is scored by reversing positively stated items and then summing up all items with a total score ranging from 0 to 40. The score indicates the degree of perceived stress; the higher the score, the more stressful the individual perceives his or her life is. Adequate reliability and validity was reported for the PSS (30), and the Cronbach's alpha for PSS in the present study was 0.85.

#### Measurement of Optimism

Optimism was assessed with the Life Orientation Test-Revised (LOT-R). The LOT-R is a self-report, 6-item questionnaire that measures generalized positive outcome expectancy or optimism (31), and has been the most commonly used instrument to measure optimism in psychological research (32). To complete the scale, respondents were asked to indicate on a 5-point scale (0 = strongly disagree and 4 = strongly agree) the extent to which they agree or disagree with each of the six items. The scale consists of three positive and three negative items, and an overall optimism score was computed by adding ratings of the positive and reversed-scored negative items, with higher scores indicating greater optimism. The LOT-R has previously shown acceptable validity and good reliability when used as a measure for optimism (33). When used in this study, it has shown moderate internal consistency with a Cronbach's alpha of 0.71.

#### Measurement of Resilience

The Resilience Scale measures the ability to cope with stress and adversity (34). In this study, we used the 14-item Resilience Scale (RS-14) to assess the resilience level of the international undergraduates. Respondents were asked to select a response on a seven-point Likert-type scale with anchoring statements from 1 (strongly disagree) to 7 (strongly agree) to indicate how well one is able to accept themselves and life in terms of adaptability, flexibility, a balanced perspective on life, determination, mastery, perseverance and so on (34). The sum of the responses on the 14 items was used to calculate the resilience scores of the participants, with higher scores indicating stronger resilience. This scale yielded a good overall reliability for different samples (34), with a Cronbach's alpha of 0.96 in the current study.

### Statistical Analysis

Analyses were conducted using SPSS statistics 23. The instruments in the study were all validated for the sample through confirmatory factor analysis and showed good fits to the data. The data was analyzed using descriptive statistics which included the means and standard deviations along with correlation coefficients. Pearson Correlation test was used to measure the correlation between the variables. An independent samples *t*-test was conducted to determine if a significant difference exists between genders.

Mediation and moderated mediation model analyses were tested using ordinary least squares path analysis and bootstrapping methods (35). Bootstrapping drew a large number of samples from the dataset and calculated the direct and the indirect effect (via perceived stress) of the independent variable (cross-cultural adaption) on the dependent variable (psychological health), which were tested against the null hypothesis that the effect was zero. The 95% confidence interval produced by the bootstrapping procedure was examined and if zero was not included within the confidence interval, the effect was considered significant (36, 37). When both the direct effect and the indirect effect were significant, partial mediating effect was proved, while full mediating effect was proved when only the indirect effect was significant (36, 37).

In addition to testing the indirect effect of cross-cultural adaption on psychological health, mediated by perceived stress, path analysis was conducted to assess whether these relationships were conditional on values of the moderators (optimism and resilience). In these analyses, cross-cultural adaption, confidence in COVID-19 control, optimism, and the cross-cultural adaption × optimism interaction term were entered as predictors of perceived stress, and cross-cultural adaption, perceived stress, optimism, resilience, the perceived stress × optimism interaction term, and the perceived stress × resilience interaction term were included as predictors of psychological health. A statistically significant interaction implied a moderating effect which was then plotted, and the significance of the slopes was examined.

In the case of a significant interaction, further analyses were conducted to probe the indirect effect by estimating conditional indirect effects at different values of the moderators (optimism and resilience). The indirect effect of cross-cultural adaption on psychological health through perceived stress was calculated at values of the moderator one standard deviation below the mean, at the mean, and above the mean (35). If the 95% bootstrapping confidence interval did not contain zero, the conditional indirect effect at that value of the moderator was considered significant (35).

All mediation and moderated mediation analyses were conducted using Hayes' PROCESS macro for SPSS, a statistical tool for path analysis-based mediation, moderation, and conditional indirect effects analyses (35). For the mediation analysis, Model 4 of PROCESS was employed to test whether perceived stress mediated the effect of cross-cultural adaption on psychological health after controlling for the confidence in COVID-19 control. For the moderated mediation analysis, Model 64 of PROCESS was used in this study. In addition, bias-corrected bootstrapping procedures with 5,000 resamples were utilized to calculate 95% confidence intervals of the effects. To avoid the problem of multicollinearity, variables were mean-centered before performing the regression. A significance level of 0.05 was used for all analyses.

## Results

### Descriptive Statistics

Means, standard deviations and correlations of the variables used in the analysis are presented in Table 1. The average GSI of the SCL-90 was 1.51, and the dimension scores based on severity of symptoms in descending order were Obsessive-Compulsive, Depression, Interpersonal Sensitivity, Paranoid Ideation, Hostility, Phobic Anxiety, Psychoticism, Anxiety, and Somatization. Cross-cultural adaption, confidence in COVID-19 control, optimism and resilience were significantly negatively correlated with GSI and all the dimension scores of the SCL-90, while perceived stress was significantly positively correlated with GSI and all the dimension scores of the SCL-90. Age was not significantly correlated with any variable.

**Table 1:** Correlations of variables in the analysis.

**Variable**	**Mean**	***SD***	**1**	**2**	**3**	**4**	**5**	**6**
1. Age	22.09	2.73						
2. Cross-cultural adaption	19.60	3.27	0.03					
3. Confidence in COVID-19 control	16.35	2.29	0.06	0.29^**^				
4. Perceived stress	16.65	6.09	−0.09	−0.31^**^	−0.29^**^			
5. Optimism	14.26	4.02	0.09	0.15^**^	0.15^**^	−0.35^**^		
6. Resilience	78.59	16.38	0.05	0.29^**^	0.20^**^	−0.29^**^	0.33^**^	
7. GSI	1.51	0.60	−0.03	−0.39^**^	−0.26^**^	0.58^**^	−0.26^**^	−0.22^**^
8. Somatization	1.31	0.50	0.02	−0.35^**^	−0.22^**^	0.46^**^	−0.21^**^	−0.22^**^
9. Obsessive-Compulsive	1.71	0.78	−0.03	−0.37^**^	−0.26^**^	0.56^**^	−0.22^**^	−0.19^**^
10. Interpersonal Sensitivity	1.60	0.74	−0.05	−0.32^**^	−0.24^**^	0.51^**^	−0.23^**^	−0.17^**^
11. Depression	1.66	0.77	−0.03	−0.40^**^	−0.26^**^	0.60^**^	−0.25^**^	−0.23^**^
12. Anxiety	1.38	0.59	0.01	−0.34^**^	−0.26^**^	0.53^**^	−0.23^**^	−0.23^**^
13. Hostility	1.47	0.65	−0.03	−0.31^**^	−0.20^**^	0.54^**^	−0.25^**^	−0.17^**^
14. Phobic Anxiety	1.42	0.62	−0.04	−0.33^**^	−0.23^**^	0.43^**^	−0.19^**^	−0.21^**^
15. Paranoid Ideation	1.58	0.72	−0.05	−0.31^**^	−0.23^**^	0.49^**^	−0.24^**^	−0.16^**^
16. Psychoticism	1.41	0.63	−0.02	−0.39^**^	−0.23^**^	0.52^**^	−0.28^**^	−0.21^**^

*SD, Standard deviation;*

** *P < 0.01*.

### Differences of Psychological Health Between Genders

The tests found no significant differences on the GSI and all the dimensions of the SCL-90 between males and females. A summary of the results is shown in Table 2.

**Table 2:** Differences of psychological health between genders.

**Psychological health**	**Gender**	**Mean**	***SD***	***t***	***P***
GSI	Male	1.50	0.59	−0.53	0.59
	Female	1.53	0.62		
Somatization	Male	1.27	0.47	−1.57	0.12
	Female	1.35	0.54		
Obsessive-Compulsive	Male	1.70	0.76	−0.42	0.67
	Female	1.73	0.80		
Interpersonal Sensitivity	Male	1.60	0.74	−0.12	0.91
	Female	1.61	0.74		
Depression	Male	1.63	0.74	−0.89	0.38
	Female	1.70	0.79		
Anxiety	Male	1.37	0.56	−0.63	0.53
	Female	1.40	0.62		
Hostility	Male	1.45	0.66	−0.41	0.68
	Female	1.48	0.64		
Phobic Anxiety	Male	1.38	0.56	−1.21	0.23
	Female	1.45	0.67		
Paranoid Ideation	Male	1.58	0.71	−0.23	0.82
	Female	1.59	0.73		
Psychoticism	Male	1.43	0.65	0.80	0.43
	Female	1.38	0.60		

*SD, Standard deviation*.

### Mediation Model Testing Results

Since age was not significantly correlated to the GSI of the SCL-90 and gender didn't yield a significant effect on the GSI of the SCL-90 in the univariate analyses, they were not controlled in the models. After controlling for the confidence in COVID-19 control, the results for the mediation model testing are presented in Table 3, from which the analysis indicated that cross-cultural adaption significantly and negatively associated with the GSI (β = −0.23, *P* < 0.01) and perceived stress (β = −0.24, *P* < 0.01), and perceived stress significantly and positively associated with the GSI (β = 0.51, *P* < 0.01). There was also a significantly negative association between confidence in COVID-19 control and perceived stress (β = −0.22, *P* < 0.01). The output showed that the direct effect of cross-cultural adaption on the GSI was significant, and the indirect effect through perceived stress on the GSI was also significant (Effect = −0.02, 95% confidence interval [−0.03, −0.01]), supporting the hypothesis (H1) that the perceived stress mediated the relationship between cross-cultural adaption and the psychological health of international medical undergraduates partially. These findings indicated that the stress perceived by the international medical undergraduates decreased when the cross-cultural adaption improved, and then the GSI decreased accordingly, which represented an improvement of the psychological health.

**Table 3:** Results of the mediation model testing.

**Path**	**β**	***SE***	***t***	***F***	***R^**2**^***
Direct effect					
Cross-cultural adaption → GSI	−0.23	0.01	−5.97^**^	140.34^**^	0.38
Perceived stress → GSI	0.51	0.01	13.07^**^		
Cross-cultural adaption → Perceived stress	−0.24	0.08	−5.32^**^	36.27^**^	0.14
Confidence in COVID-19 control → Perceived stress	−0.22	0.12	−4.85^**^		
Indirect effect	Effect	*SE*	*LLCI*	*ULCI*	
Cross-cultural adaption → Perceived stress → GSI	−0.02	0.01	−0.03	−0.01	

*β, standardized coefficient; SE, standard error; LLCI, lower level of the confidence interval; ULCI, upper level of the confidence interval;*

** *P < 0.01*.

### Moderated Mediation Model Testing Results

**Figure 2:**
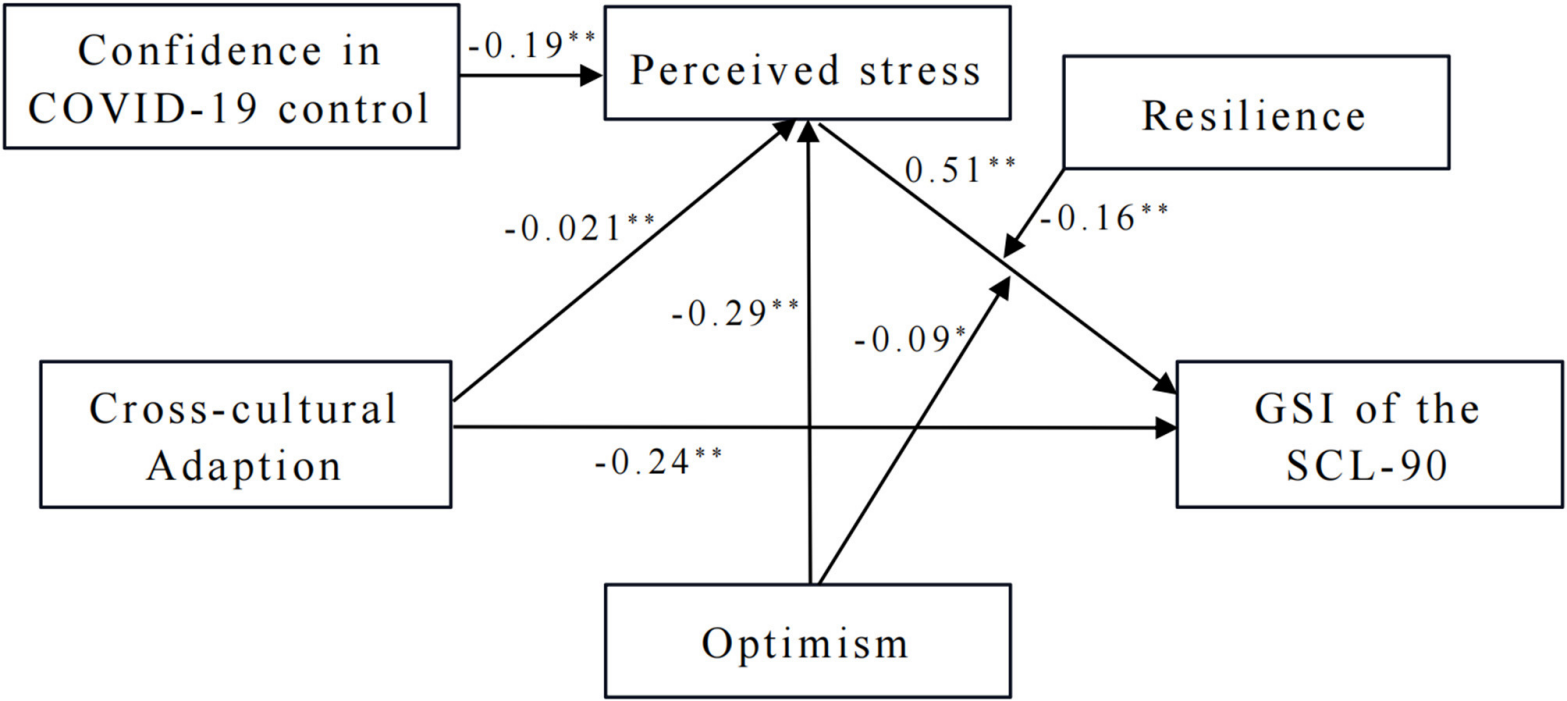
The final model with statistic values. *P < 0.05, **P < 0.01.

The results for the moderated mediation model testing are shown in Table 4, and the final model with statistic values is shown in Figure 2. Unfortunately, optimism did not moderate the relationship between cross-cultural adaption and perceived stress, as the cross-cultural adaption × optimism interaction term was not significant. However, it had a significant and negative effect (β = −0.29, *P* < 0.01) on perceived stress directly, whose strength was even stronger than the direct effect of cross-cultural adaption (β = −0.21, *P* < 0.01) and confidence in COVID-19 control (β = −0.19, *P* < 0.01) on perceived stress. Combination of the cross-cultural adaption, confidence in COVID-19 control and optimism explained 22% of the variance (*R*^2^ = 0.22, *F* = 31.30, *P* < 0.01) in perceived stress.

**Table 4:** Results of the moderated mediation model testing.

**Variable**	**β**	***SE***	***t***	***F***	***R^**2**^***
Independent variable: Perceived stress				31.30^**^	0.22
Cross-cultural adaption	−0.21	0.08	−4.76^**^		
Confidence in COVID-19 control	−0.19	0.12	−4.30^**^		
Optimism	−0.29	0.06	−6.75^**^		
Cross-cultural adaption × Optimism	−0.01	0.02	−0.01		
Independent variable: GSI				57.04^**^	0.43
Cross-cultural adaption	−0.24	0.02	−6.16^**^		
Perceived stress	0.51	0.01	12.66^**^		
Optimism	−0.06	0.02	−1.56		
Resilience	0.03	0.01	0.86		
Perceived stress × Optimism	−0.09	0.01	−2.16*		
Perceived stress × Resilience	−0.16	0.01	−4.00^**^		

β, standardized coefficient; SE, standard error; *P < 0.05,

** *P < 0.01*.

In the moderated mediation model, the strength of the relationship between perceived stress and the GSI (β = 0.51) was stronger than the relationship between cross-cultural adaption and the GSI (β = −0.24). Although optimism and resilience did not significantly associate with the GSI, both interactions with perceived stress were significant. The combination of the variables above explained 43% of the variance in psychological health of international medical undergraduates.

**Figure 3:**
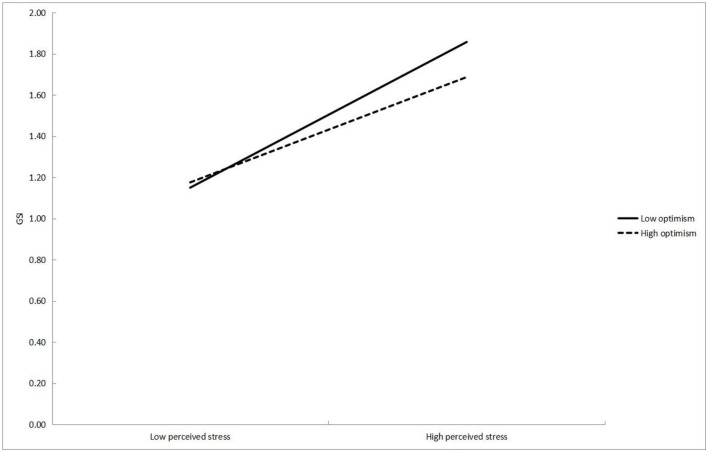
The moderation of optimism on the relationship between perceived stress and GSI of the SCL-90.

**Figure 4:**
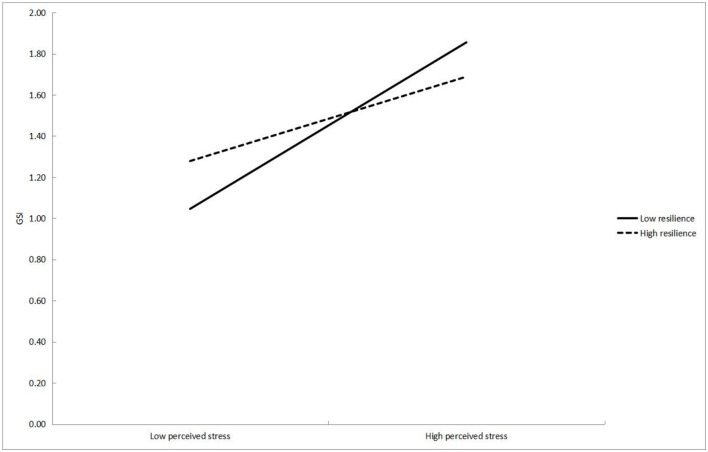
The moderation of resilience on the relationship between perceived stress and GSI of the SCL-90.

The interaction between perceived stress and optimism (β = −0.09, *P* < 0.05), as well as the interaction between perceived stress and resilience (β = −0.16, *P* < 0.01), were all negative on the GSI, indicating that optimism and resilience negatively moderated the relationship between perceived stress and the GSI, supporting H3 and H4. In order to interpret the interaction term, the simple slopes of optimism and resilience predicting the GSI are presented in Figures 3, 4. For those who were low in optimism, higher perceived stress was associated with higher GSI (simple slope = 0.06, *t* = 11.48, *P* < 0.01). However, for those who were high in optimism, the association was weakened (simple slope = 0.04, *t* = 7.93, *P* < 0.01), which means optimism dampens the positive relationship between perceived stress and the GSI. Thus, when there is a high level of perceived stress with a high level of optimism, it is less likely to have a serious negative impact on psychological health. Similarly, the slope of the regression line of perceived stress predicting the GSI at low (simple slope = 0.07, *t* = 10.79, *P* < 0.01) and high (simple slope = 0.03, *t* = 6.02, *P* < 0.01) level of resilience were all significant, but the slope was smaller when the level of resilience was high. Therefore, resilience mitigated the positive relationship between perceived stress and the GSI, too.

Moderation of the indirect effect of cross-cultural adaption on the GSI was tested for low, medium and high levels of optimism, and at the corresponding levels of resilience as well. As shown in Table 5, the indirect effects were significant for all the levels of optimism and resilience but differentiated at different levels. When optimism or resilience moved to higher levels, the indirect effect was seen to be smaller, which means that the indirect effect of cross-cultural adaption on the GSI via perceived stress becomes weaker as optimism or resilience improves. The high optimism and resilience subgroup was affected the least compared with other subgroups by the indirect effect of cross-cultural adaption associated with the GSI of the SCL-90.

**Table 5:** Conditional indirect effects of cross-cultural adaption on psychological health at different levels of optimism and resilience.

**Level of optimism**	**Level of resilience**	**Indirect effect**	***SE***	***LLCI***	***ULCI***
−1*SD*	−1*SD*	−0.029	0.01	−0.05	−0.01
−1*SD*	Mean	−0.022	0.01	−0.04	−0.01
−1*SD*	+1*SD*	−0.016	0.01	−0.03	−0.01
Mean	−1*SD*	−0.026	0.01	−0.04	−0.01
Mean	Mean	−0.020	0.01	−0.03	−0.01
Mean	+1*SD*	−0.013	0.01	−0.02	−0.01
+1*SD*	−1*SD*	−0.024	0.01	−0.04	−0.01
+1*SD*	Mean	−0.017	0.01	−0.03	−0.01
+1*SD*	+1*SD*	−0.010	0.01	−0.02	−0.01

*SE, standard error; LLCI, lower level of the confidence interval; ULCI, upper level of the confidence interval*.

## Discussion

The COVID-19 pandemic adversely impacts on the psychological health of university students worldwide. However, it's still possible that individuals can adjust to the pandemic through remaining positive (38). In our study, the average GSI of the SCL-90 among international medical undergraduates was mild (1.51), which is similar to that of previous studies in China (39, 40). The possible reason may be related with a more stable psychological reaction that the medical undergraduates had, due to the knowledge and training from campus.

Our study proposed a mediation model first to identify the relationship between cross-cultural adaption and psychological health among international medical undergraduates during the COVID-19 pandemic, and to see whether the relationship was mediated by perceived stress. We found that cross-cultural adaption significantly and negatively associated with the GSI of SCL-90 (β = −0.23, *P* < 0.01) and perceived stress (β = −0.24, *P* < 0.01) with moderate effects; and perceived stress significantly and positively associated with the GSI of SCL-90 (β = 0.51, *P* < 0.01) with a strong effect. Consistent with previous studies, our findings support the positive relationship between cross-cultural adaption and psychological health among international medical undergraduates, which implies the students with better cross-cultural adaption adjust better to the new environment and are optimally functioning in social arenas (41–43). Furthermore, our findings revealed that in addition to the significant direct effect on the GSI of SCL-90, cross-cultural adaption also had a significant indirect effect on the GSI of SCL-90 by influencing the stress perceived by international medical undergraduates. This means that perceived stress partially mediated the relationship between cross-cultural adaption and psychological health among international medical undergraduates, which was in line with the previous research findings among immigrant and other populations (44, 45). Consequently, international medical undergraduates with poor cross-cultural adaption were likely to perceive more stress, which was associated with increase of GSI in the current sample. Since perceived stress had a strong effect on psychological health, how to manage the stress effectively would be crucial for international medical undergraduates.

In the final moderated mediation model, our results revealed that although no moderating effect of optimism was found on the relationship between cross-cultural adaption and stress perceived by international medical undergraduates, optimism had a negative direct effect on perceived stress (β = −0.29, *P* < 0.01), which should also be considered as a useful resource to combat stress. Previous studies have proven that optimism could influence stress-perception (46, 47), and the individuals who had higher level of optimism, as a consequence of the optimistic style of appraisal, were more likely to report active ways of coping with stressful situations, which in turn led to perceive less stress than those with lower level of optimism (48). Therefore, optimism is a protective factor of perceived stress for international medical undergraduates. Importantly, similar to one previous research finding (49), our study also revealed that the confidence in COVID-19 control was negatively correlated with perceived stress of international medical undergraduates (β = −0.19, *P* < 0.01). During the COVID-19 pandemic period, although the pandemic was a stressor for international medical undergraduates, the achievements in combating COVID-19 across different countries might encourage them to cooperate and abide by the various control measures, and their confidence in COVID-19 control might increase accordingly, which could alleviate the perception of stress (50). This finding suggests that during the pandemic of COVID-19, building confidence in COVID-19 control in the students may be a good strategy to make international medical undergraduates feel less stress and improve their psychological health. Therefore, the improvement of optimism and confidence in COVID-19 control could help international medical undergraduates to perceive less stress.

Moreover, in our moderated mediation model, optimism and resilience negatively moderated the relationship between perceived stress and the GSI of SCL-90, and the effects were differentiated for subgroups, which implied that the indirect effect of cross-cultural adaption on psychological health via perceived stress was dependent on the levels of optimism and resilience. Our findings showed that the indirect effect of cross-cultural adaption on psychological health via perceived stress was weaker for international medical undergraduates with high level of optimism and resilience compared with those with low or medium level of optimism and resilience. These findings are also in agreement with previous studies which revealed that higher level of optimism and resilience, as internal resources, would be valuable strategies to improve coping mechanisms for stress on psychological health (21, 51–53). This indicates that an intervention of protection might be useful for international medical undergraduates, in particular for those with low level of optimism and resilience. Various positive psychology exercises have demonstrated that optimism and resilience could be taught, learned and developed over time (21, 54, 55). Findings of our study provide evidence for developing training programs that support fostering high levels of optimism and resilience. In this sense, we suggest this knowledge could be used by university educators and managers to build positive characteristics and improve strengths for international undergraduates under the circumstances of COVID-19 pandemic.

This study has several limitations. First of all, since our study population only included the international undergraduates at one medical university of China, caution should be noted in generalizing the findings from this study to other international undergraduates. This warrants a replication of this study inquiry across a number of universities in China before more robust conclusions can be drawn. Second, the data was obtained by self-report measurements and could have included both participant bias and dishonesty, so it is possible that data could be inaccurate. Third, due to the cross-sectional study design, causal inferences cannot be made regarding the relationships between variables in this study. A possible future research opportunity is to investigate through longitudinal approach study. Fourth, as only two integral components of psychological capital i.e., optimism and resilience are studied, other aspects of the psychological capital should be taken into account in future studies.

Despite the limitations, our study offers some positive findings and opportunities for further research in the area of psychological health of international medical undergraduates and enriches the literature of cross-cultural adaption and positive psychology in higher education research. Our findings are also potentially useful for psychological health intervention programs for international medical undergraduates to cope with stressful situations such as COVID-19 pandemic more effectively. Although the results and recommendations from this study cannot eliminate the stressors, they may help lessen the degree to which the international medical undergraduates are affected by the poor cross-cultural adaption. Since previous studies have developed a series of brief micro-training interventions that focused on improving individuals' optimism and resilience (56–58), it is worth implementing among the international medical undergraduates to counteract stress induced by cross-cultural adaption, and maintain their psychological health during the COVID-19 pandemic. University educators ought to promote and make use of programs such as the ones that cope with stress and boost optimism and resilience in order to support students not only adapt well to a new culture, but also keep good psychological health.

## Conclusion

In the present study, we found a positive association between cross-cultural adaption and psychological health among international medical undergraduates, which was mediated by perceived stress. Optimism and confidence in COVID-19 control were negatively correlated with perceived stress. In addition, optimism and resilience negatively moderated the indirect effect of cross-cultural adaption on psychological health through perceived stress. Therefore, collaborations from multiple departments on campus are required to implement the training programs that cope with stress and foster high levels of optimism and resilience, in order to maintain psychological health among international medical undergraduates in China during the COVID-19 pandemic.

## Data Availability Statement

The raw data supporting the conclusions of this article will be made available by the authors, without undue reservation.

## Ethics Statement

The studies involving human participants were reviewed and approved by the Research Ethics Committee at China Medical University. The patients/participants provided their written informed consent to participate in this study.

## Author Contributions

XiW drafted the manuscript. LL helped analyzing the statistics. XuW and MQ collected the data. LY and YG helped with the study design and data analysis. BP provided the guidance of the study design and reviewed the manuscript. All authors contributed to the article and approved the submitted version.

## Conflict of Interest

The authors declare that the research was conducted in the absence of any commercial or financial relationships that could be construed as a potential conflict of interest.

## Publisher's Note

All claims expressed in this article are solely those of the authors and do not necessarily represent those of their affiliated organizations, or those of the publisher, the editors and the reviewers. Any product that may be evaluated in this article, or claim that may be made by its manufacturer, is not guaranteed or endorsed by the publisher.
